# Accelerated Discovery of 3D Printing Calcium Sulphoaluminate Cement Composites Using Data-Driven Multi-Objective Optimization

**DOI:** 10.3390/ma19132842

**Published:** 2026-07-03

**Authors:** Yan Liu, Qichang Fan, Yuanyuan Zheng, Dan Meng, Zhen Zhao, Shibo Wang, Longhao Gu

**Affiliations:** 1 School of Architectural Engineering, Qingdao Agricultural University, Qingdao 266033, China; liuyan@qau.edu.cn (Y.L.); zhaozhen@qau.edu.cn (Z.Z.); gulonghao@qau.edu.cn (L.G.); 2 State Key Laboratory for Tunnel Engineering, School of Civil Engineering, Sun Yat-Sen University, Zhuhai 519082, China; fanqch5@mail2.sysu.edu.cn; 3 Southern Marine Science and Engineering Guangdong Laboratory (Zhuhai), Zhuhai 519082, China

**Keywords:** additive manufacturing, data-driven multi-objective optimization, calcium sulphoaluminate cement composites, accelerated discovery

## Abstract

Additive manufacturing enables the fabrication of complex geometries and structures that are difficult to attain by conventional methods. However, many printable materials exhibit inherent trade-offs among their performance properties. Traditional material design, often relying on intuition-driven and inefficient trial-and-error approaches, frequently fails to identify optimal formulations. In this study, we propose a machine learning-assisted framework to efficiently refine the composition of 3D-printable calcium sulphoaluminate (CSA) cement composites with a balanced trade-off among thixotropy, mechanical strength, and shape stability. Our approach integrates a multi-objective optimization algorithm with a data-driven surrogate model to intelligently propose new mix proportions, thereby reducing the number of required experiments. Starting from seven primary formulations and 28 initial experimental samples, the method identified 23 improved mix proportions after only 20 algorithm iterations. The workflow demonstrates how optimization-assisted formulation refinement can accelerate the search for better-performing materials and is potentially adaptable to other material design challenges.

## 1. Introduction

Additive manufacturing enables the fabrication of complex geometries and structures that are difficult to attain by conventional methods. Recently, 3D printing has gained adoption in civil and structural engineering owing to its design freedom and construction efficiency [1,2,3,4,5]. Unlike conventional construction, 3D printing extrudes material layer by layer, eliminating formwork [6,7]. However, the development of new printable materials still relies on empirical knowledge and extensive trial and error, limiting efficiency and scalability. For cementitious materials, a balance of mechanical strength, thixotropic behavior, and shape retention is required [8,9,10]. Traditional strategies often focus on a single performance metric [11], leading to material waste and suboptimal outcomes. To promote wider adoption, the accelerated development of high-performance, multi-property material systems is essential [12].

Recent studies have introduced autonomous systems for material discovery [13,14,15,16,17,18,19,20,21,22]. For example, Gongora et al. [13] used Bayesian optimization to identify structures with high compressive toughness. Rizkin et al. [14] integrated microfluidic reactors with Latin hypercube sampling to screen catalysts. Despite these advances, most work remains single-objective, and experimental runs are costly and time-consuming. Data-efficient multi-objective optimization methods that strategically sample the design space offer a promising pathway to reduce experimental effort [23].

**Figure 1:**
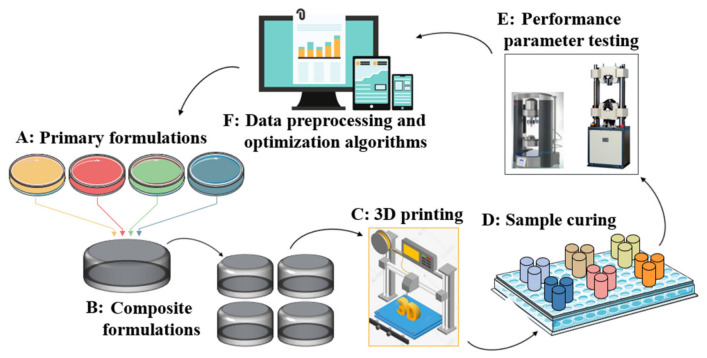
Schematic workflow of proposed data-driven framework: (**A**) Primary formulations for dispensing desired formulations. (**B**) Composite formulations. (**C**) 3D printer for sample fabrication. (**D**) Sample standard environmental curing. (**E**) 3D-printed sample multi-performance testing. (**F**) Non-dominated sorting whale optimization algorithm for formulation and performance evaluation, as well as suggestion for which new formulations to test.

In this study, we introduce a data-driven framework (Figure 1) to efficiently refine the composition of 3D-printable calcium sulphoaluminate (CSA) cement composites with balanced thixotropy, strength, and shape stability. The framework integrates a multi-objective optimization algorithm with a surrogate model to intelligently propose new mix proportions, reducing required experiments. Starting from seven primary formulations and 28 initial samples, the method identified 23 improved mix proportions after only 20 algorithm iterations.

The proposed workflow begins by blending primary formulations in designated ratios (Figure 1A) and homogenizing the mixture thoroughly (Figure 1B) to produce various composite materials. Each resulting formulation is then transferred to a 3D printer for sample fabrication (Figure 1C), followed by a curing stage (Figure 1D) to finalize specimen preparation. The samples undergo performance testing to quantify key quantitative metrics, including thixotropy, compressive strength, and shape stability (Figure 1E). To efficiently allocate experimental resources and accelerate the identification of superior material designs, this work implements a data-driven strategy based on the non-dominated sorting whale optimization algorithm (NSWOA) (Figure 1F). This algorithm iteratively learns from completed experiments to recommend subsequent formulations for testing, balancing the exploitation of high-performing candidates with the exploration of under-evaluated regions in the design space. The workflow repeats until the predefined experimental budget is exhausted.

Through only 20 algorithmic iterations, we rapidly explored and expanded the performance space, identifying 23 optimized mix proportions that exhibit optimal trade-offs among competing properties. This approach is potentially adaptable to a wide range of formulation design challenges in materials science.

The genuine novelty of this work is threefold: (i) the first application of the NSWOA to CSA cement composites, (ii) the integration of bootstrap ensemble uncertainty quantification with active learning, and (iii) the experimental validation of 23 Pareto-optimal formulations, which goes beyond typical in silico studies.

## 2. 3D Printing Materials and Performance Testing Methods

### 2.1. Base Ingredients and Material Formulations

Our approach begins with the preparation of a series of mutually compatible cement mortar base formulations—comprising cement, water, ultrafine quartz sand (UQS), and a water-reducing agent (WRA)—designed to exhibit a diverse range of mechanical, rheological, and stability properties [24,25]. These constituents are widely employed in mortar-based 3D printing processes.

Rather than developing printing materials from scratch, we expanded these four fundamental constituents by incorporating eight commercially available functional ingredients—one thickener (HPMC, hydroxypropyl methyl cellulose), four rheology modifiers (diatomite; bentonite; metakaolin; PMS, paper milling sludge), two retarders (TA, tartaric acid; SG, sodium gluconate), and one fiber (PP, polypropylene). From this library of twelve components, we formulated seven primary mixtures (designated I to VII) to serve as the foundational compositions for subsequent optimization, as detailed in Table 1.

All primary formulations were designed within a suitable viscosity range to ensure the printability of any potential composite mixture. The primary cementitious component was CSA cement (grade 42.5) [26], with its chemical composition provided in Table 2. UQS was incorporated to enhance compressive strength and shape stability, while HPMC served as a thickener to improve interlayer bonding [10]. Rheological control was achieved using bentonite, diatomite, metakaolin, and PMS as modifiers to increase the paste’s yield stress and stiffness [26,27]. TA and SG were employed as retarders to enable adjustable control over workable time. A polycarboxylate-based WRA was added to improve flowability, and PP fibers (aspect ratio of 120) were included as reinforcement. The physical properties of PP fibers are summarized in Table 3.

These seven primary formulations (I–VII) were pre-selected based on their distinct printability, viscosity, and unique mechanical, rheological, and stability characteristics. The material library could be readily expanded to suit different printing techniques or applications. Composite formulations were generated by blending the selected primaries in proportions determined by the optimization algorithm, producing a series of mortars with varied compositions for evaluation.

### 2.2. 3D Printing and Performance Testing

**Figure 2:**
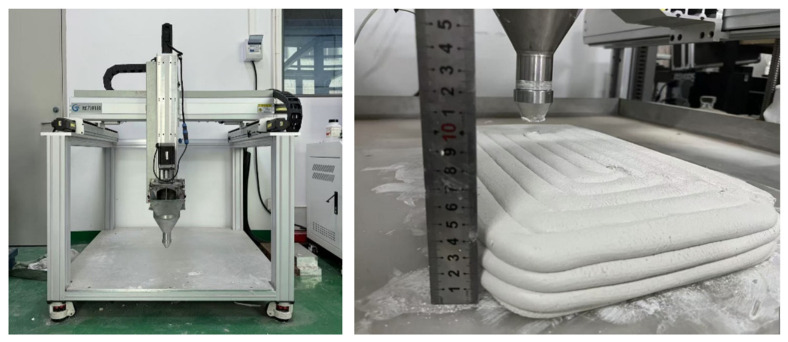
Structure of 3D printer and printed sample.

All specimens were fabricated using a lab-scale 3D printer (Pottery Artist, Dianfeng Company, Yueyang, China), as illustrated in Figure 2. The printer was configured with a screw mixing mechanism, and the material loading and printing speeds were set to 15 mm/s and 10 mm/s, respectively. After the mortar was thoroughly mixed, the rheological properties of the fresh mortar were characterized, followed by an evaluation of the shape stability and mechanical performance of the printed samples. Printed specimens measured 5 cm × 25 cm × 70 cm. The test results are summarized in Table 1, and detailed testing procedures are described below.

#### 2.2.1. Thixotropy

To characterize rheological behavior, Qian et al. [28] proposed an indicator, which is called the structural parameter, and it represents the relationship between the static and dynamic yield stresses. The higher the structural parameters are, the better the thixotropic property will be. And its calculation is given by Equation (1).

(1) $$I_{thix}=\tau_{i}/\tau_{e}$$

where $I_{thix}$ represents the structure parameters; $\tau_{i}$ and $\tau_{e}$ are the maximum shear stress and shear stress equilibrium value, respectively.

#### 2.2.2. Mechanical Strength

Compressive strength was measured after 28 days of curing using a universal testing machine (Model C43.504, MTS, Eden Prairie, MN, USA) with a load capacity ranging from 10 N to 50 kN. Tests were performed on 20 mm cubic specimens at a loading speed of 1 mm/min. For each formulation, a minimum of six specimens were tested to ensure representative strength values.

#### 2.2.3. Structure Deformation

Structural deformation, quantified as the average maximum deformation measured from three directions, was used to assess the geometric quality and printing accuracy of fabricated specimens. The deformation value was calculated using Equation (2).

(2) $$D=\left[\frac{\left|l-l_{0}\right|}{3l_{0}}+\frac{\left|w-w_{0}\right|}{3w_{0}}+\frac{\left|h-h_{0}\right|}{3h_{0}}\right]$$

where *D* represents structure deformation; *l*, *w* and *h* are the stable length, width and height of 3D-printed mortars after extrusion; *l*_0_, *w*_0_, and *h*_0_ are the length, width and height of the model, respectively.

## 3. Data-Driven Multi-Objective Optimization

### 3.1. Surrogate Model: Bayesian-Optimized BP Neural Network

3D-printed mortar is an intrinsically complex multiphase composite material influenced by multiple interacting factors. The relationship between its mix proportions and macro-properties is highly nonlinear and uncertain, rendering the development of an accurate explicit mathematical model challenging. To address this, the present study constructs a BP neural network optimized by Bayesian optimization to serve as a surrogate model. Bayesian optimization is specifically designed for black-box problems with high evaluation costs, enabling the efficient approximation of the global optimum with minimal iterations [29]. It also provides point predictions and uncertainty estimates, aligning with the demands of material performance prediction [30,31].

**Figure 3:**
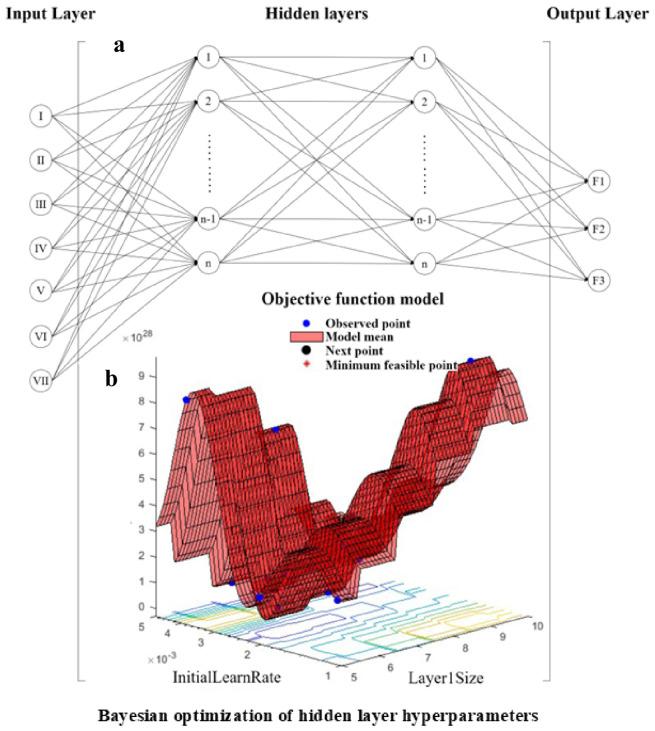
A schematic diagram of the Bayesian-optimized BP neural network (BO-BP) serving as the surrogate model. The network consists of an input layer (seven nodes, corresponding to the proportions of the seven primary mixtures), two hidden layers (with batch normalization and ReLU activation), and an output layer (three nodes, predicting the structure parameter, compressive strength, and deformation). Dropout is applied after the first hidden layer for regularization. The architecture and hyperparameters (learning rate, regularization coefficients) are determined by Bayesian optimization using 5-fold cross-validation on the initial dataset. (**a**) Architecture of the prediction algorithm; (**b**) Actual prediction results.

An initial dataset of 28 data points was constructed, comprising the seven primary formulations and 21 additional composite samples systematically designed based on the seven initial formulations. All samples were experimentally characterized for three target performance values (Table 4). A neural network with an input layer of seven neurons (mix proportions) and an output layer of three neurons (performance indicators) was employed. This network is referred to as the BO-BP (Bayesian-optimized BP) surrogate model. Given the limited sample size, Bayesian optimization was used for hyperparameter tuning, searching 1–2 hidden layers and 5–10 neurons per layer. Fivefold cross-validation and L2 regularization were applied to mitigate overfitting. The final architecture is illustrated in Figure 3.

### 3.2. Uncertainty Quantification via Bootstrap Ensemble

**Figure 4:**
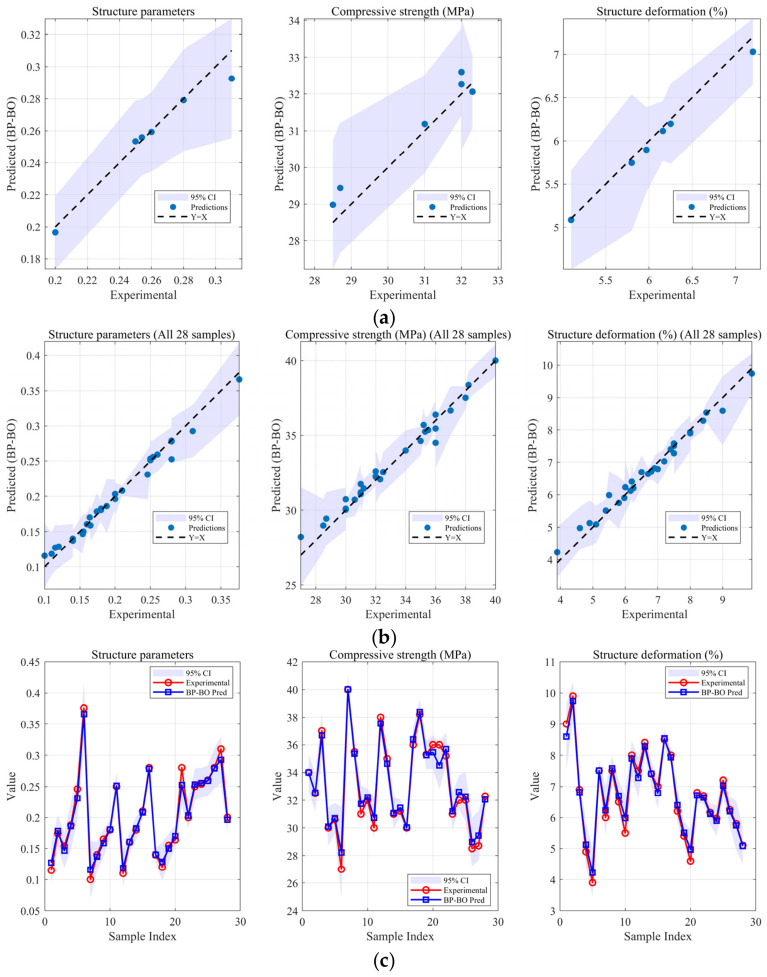
The uncertainty quantification of the BO-BP surrogate using a bootstrap ensemble (20 sub-networks). (**a**) Predicted vs. actual values on the test set (6 samples). Each point is the mean prediction of the ensemble; the light blue band represents the 95% confidence interval (mean ± 1.96 × std). Most points lie within the band and close to the diagonal Y = X, indicating good generalization. (**b**) The same goes for all 28 samples (training + test). The confidence bands for the structure parameter and compressive strength are narrow, reflecting high model confidence; the band for deformation is slightly wider, indicating higher predictive uncertainty. (**c**) Predictions and 95% confidence bands are plotted against the sample index (1–28). The training set (samples 1–22) shows very narrow bands; the test set (samples 23–28) exhibits slightly wider intervals, as expected for unseen data. All test points are covered by the confidence intervals, validating the reliability of the bootstrap-based uncertainty estimates.

To estimate the prediction uncertainty of the BO-BP surrogate, we employ a bootstrap ensemble [32]. From the original training set (22 samples), we draw 20 bootstrap samples with replacement, each of the same size. A separate BO-BP network is trained on each bootstrap sample, producing an ensemble of 20 sub-networks. For any input, the ensemble outputs a set of predictions; the mean is taken as the point prediction, and the 95% confidence interval is computed as mean ± 1.96 × standard deviation. This quantifies the epistemic uncertainty arising from limited training data. The results are presented in Figure 4.

### 3.3. Multi-Objective Optimization: NSWOA

After constructing the surrogate model, we apply the non-dominated sorting whale optimization algorithm (NSWOA) to search for optimal mix proportions. Originally proposed by Jangir et al. [33,34], the NSWOA integrates the whale optimization algorithm with the multi-objective handling strategy of NSGA-II [35]. It offers fast convergence and good solution diversity [36,37,38,39].

The optimization procedure is as follows:

Step 1: Parameter and population initialization—Decision variables are the proportions of seven components (sum to 1). Objectives: The objectives are to maximize the structure parameter and compressive strength and minimize deformation. A population of 100 feasible solutions is initialized.

Step 2: Non-dominated sorting and crowding distance—Pareto fronts are identified, and crowding distance is calculated to preserve diversity.

Step 3: Position update—Each individual updates its position using encircling, bubble-net, or random search according to the WOA mechanism.

Step 4: Elite preservation—Parent and offspring populations are merged and non-dominated-sorted, and the best individuals (based on rank and crowding distance) are selected.

Step 5: Termination and output—Steps 2–4 are repeated until 100 generations. The first non-dominated front is output as the Pareto-optimal set.

**Figure 5:**
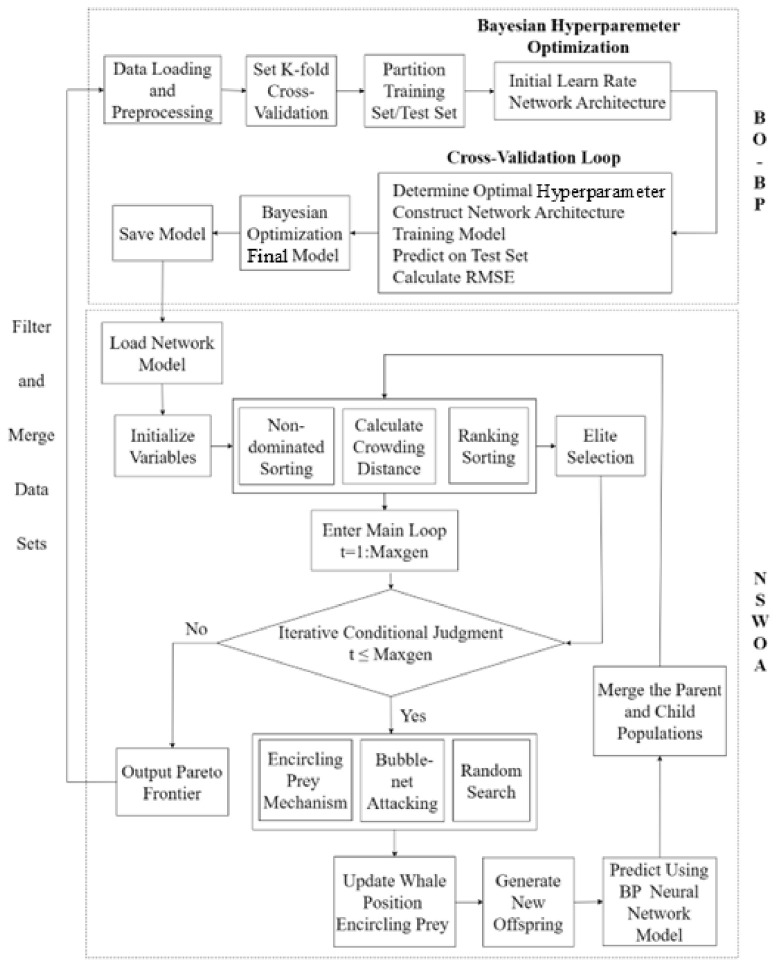
A flowchart of the two-stage data-driven optimization framework. Stage 1: Surrogate model construction. Starting from 28 initial experimental samples, Bayesian optimization with 5-fold cross-validation tunes the architecture (number of hidden layers, neurons per layer, learning rate) and hyperparameters (L2 regularization, dropout rate) of a BP neural network. The final BO-BP model serves as a fast surrogate for predicting thixotropy, strength, and deformation. Stage 2: Multi-objective optimization using the NSWOA. The algorithm iteratively searches the 7-dimensional composition space (summation constraint = 1) to maximize the structure parameter and compressive strength while minimizing deformation. At each generation, non-dominated sorting and crowding distance calculation are performed to maintain diversity. The Pareto-optimal formulations are extracted every iteration, and the most promising ones (7 per iteration) are selected for experimental validation. New experimental data are fed back to update the surrogate model, closing the active learning loop. The process repeats for 20 iterations, after which the final Pareto set is reported.

This workflow is illustrated in Figure 5.

### 3.4. Optimization Results and Composition Analysis

To experimentally validate the reliability of the algorithm’s predictions, five formulations were randomly selected from the Pareto front for testing. Table 5 presents the mix proportions, predicted values, measured values, and relative errors. The results confirm the high predictive accuracy of the model.

**Figure 6:**
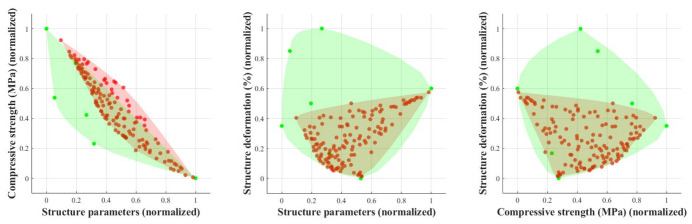
An exploration of the performance space before and after 20 iterations. The green region represents the space covered by the seven initial formulations (28 samples) and the red region the space covered by the 140 algorithm-generated formulations. All objectives are normalized to [0, 1]. The red region expands significantly toward higher strength and lower deformation, demonstrating that the algorithm discovers previously unexplored high-performance areas.

Beyond identifying superior mix proportions, the algorithm demonstrates its exploration capability within the performance space, effectively extending the boundaries towards higher strength and lower deformation (Figure 6).

**Figure 7:**
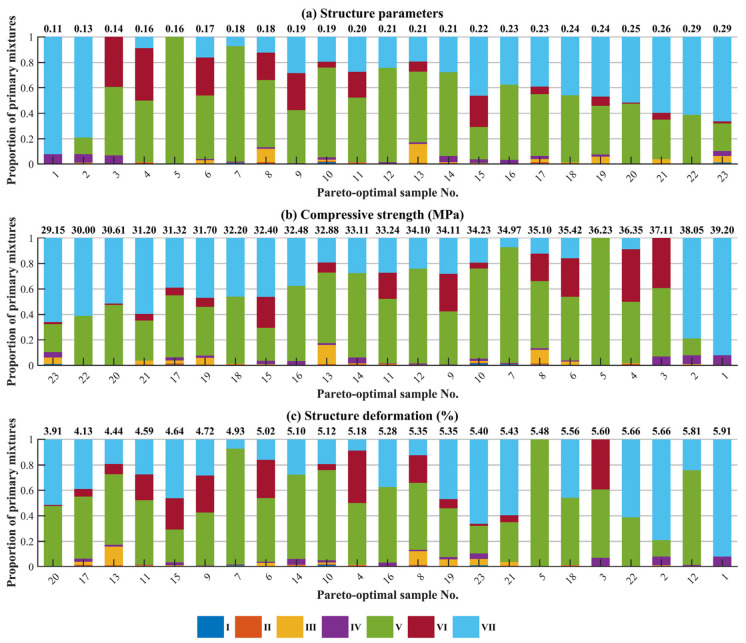
The composition of the 23 Pareto-optimal formulations. Each stacked bar represents one formulation (sample numbers 1–23). The three subplots sort the formulations by (**a**) structure parameter (descending), (**b**) compressive strength (descending), and (**c**) structural deformation (ascending). Numbers above bars indicate objective values. High-performing formulations contain very low amounts of retarder-rich mixtures (I, II) and higher amounts of bentonite- and PP fiber-rich mixtures (V, VII).

The Pareto-optimal set was further examined from a chemical composition perspective to elucidate the relationship between sample performance and formulation design. As shown in Figure 7, the algorithm consistently favors formulations with very low proportions of retarder-rich mixtures (I and II). This can be attributed to the presence of tartaric acid (TA) and sodium gluconate (SG) in these mixtures, which delay cement setting time and consequently increase structural deformation. In contrast, higher proportions of Mixtures V and VII appear in the best-performing samples. The bentonite in Mixture V enhances thixotropy, contributing to improved structural parameters and reduced deformation. The polypropylene (PP) fibers in Mixture VII further increase compressive strength. This trend confirms that the algorithm effectively learns to minimize retarder-rich components and favor bentonite- and PP fiber-rich formulations, while Mixture VI—which contains TA and paper milling sludge—is carefully balanced to leverage its high thixotropy without compromising strength and deformation.

## 4. Comparative Analysis of Algorithms

### 4.1. Surrogate Model Comparison

**Figure 8:**
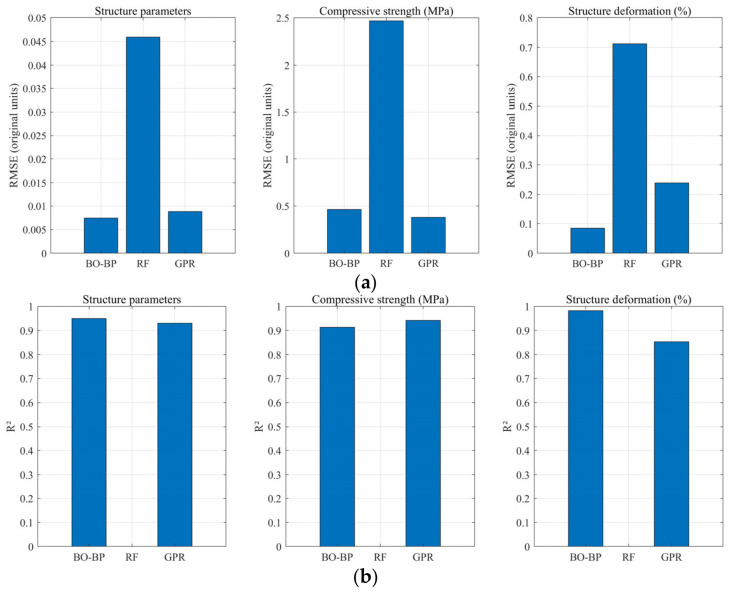
A performance comparison of the three surrogate models (BO-BP, GPR, RF) on the test set (6 samples). (**a**) The RMSE in original units. (**b**) The coefficient of determination (R^2^). BO-BP achieves the highest R^2^ for the structure parameter (0.950) and structure deformation (0.982), while GPR performs slightly better for compressive strength (R^2^ = 0.942 vs. 0.913). RF yields negative R^2^ values, indicating poor generalization.

To select the most appropriate surrogate model, we compared the BO-BP network with Gaussian Process Regression (GPR) and Random Forest (RF) using the same 5-fold cross-validation (Figure 8). Table 6 summarizes the RMSE and R^2^ on the test set (six samples). BO-BP achieves the best performance for the structure parameter (R^2^ = 0.950) and structural deformation (R^2^ = 0.982), while GPR performs slightly better for compressive strength (R^2^ = 0.942 vs. 0.913). RF yields negative R^2^ values, indicating poor generalization.

Therefore, BO-BP is selected as the primary surrogate model due to its superior performance in two objectives and its built-in uncertainty quantification (Figure 4).

### 4.2. Optimization Algorithm Comparison

**Figure 9:**
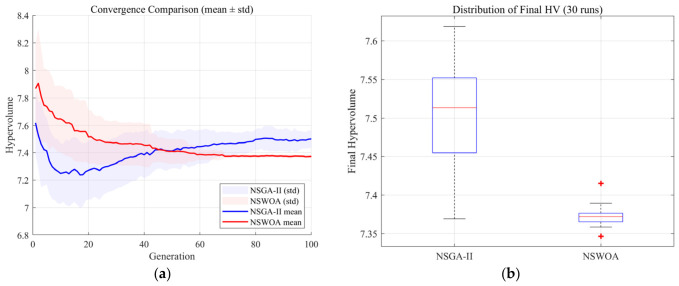
The convergence behavior of the NSWOA and NSGA-II over 30 independent runs (population size = 100; generations = 100). (**a**) The mean hypervolume (HV) ± standard deviation (shaded bands) as a function of generation. The NSWOA (red) converges rapidly, reaching a HV comparable to NSGA-II’s final value in only 20 generations. (**b**) A boxplot of the final HV values (generation 100). NSGA-II shows a higher median but larger variance, whereas the NSWOA is more consistent. The Wilcoxon rank-sum test yields *p* = 1.94 × 10^−10^, indicating a significant difference at generation 100, though for short-horizon optimization (≤20 generations), the NSWOA is clearly superior.

To evaluate the convergence efficiency of the two multi-objective optimizers, we performed 30 independent runs of the NSWOA and NSGA-II and recorded the hypervolume (HV) at generations 10, 20, 30, and 100. The results are summarized in Table 7 and visualized in Figure 9. A striking observation is that the NSWOA reaches a mean HV of 7.5147 after only 20 generations, while NSGA-II attains a mean HV of 7.4998 after 100 generations—a virtually identical solution quality with only one-fifth of the iterations. At generation 20, the NSWOA significantly outperforms NSGA-II (7.5147 vs. 7.2713, *p* = 6.36 × 10^−5^). After 100 generations, NSGA-II yields a marginally higher HV (7.4998 vs. 7.3722, *p* < 0.01). Notably, the NSWOA exhibits much smaller standard deviations at later generations (e.g., 0.0119 at generation 100 vs. 0.0624 for NSGA-II), indicating more consistent convergence.

Therefore, for the present study where only 20 algorithmic iterations were performed within the active learning loop, the NSWOA is the most appropriate choice. It successfully identified 23 Pareto-optimal formulations with a solution quality that rivals what NSGA-II would achieve after 100 iterations while requiring only one-fifth of the iterations.

In addition to convergence efficiency, we also compared the prediction accuracy of the formulations suggested by the two optimizers. To further assess the practical reliability of the surrogate model, we extracted four Pareto-optimal formulations from the final front of the NSWOA (generation 20) and four from NSGA-II (generation 30) and experimentally tested them. Table 8 presents the mix proportions, the predicted performance values, the experimentally measured values, and the relative errors. The average prediction error for the NSWOA-selected formulations is below 1.0%, while that for the NSGA-II-selected formulations is around 15.7% (with large variability). This indicates that the surrogate model trained within the NSWOA-driven active learning loop achieves higher predictive accuracy on the formulations it proposes, likely because the model has seen similar compositions during iterative sampling. Nonetheless, both sets of predictions are within acceptable engineering tolerance (except one NSGA-II sample with 26.5% error). These results further support the choice of the NSWOA as the optimizer, as it not only converges faster but also yields formulations that are more accurately predicted by the surrogate, reducing the risk of experimental surprises.

## 5. Conclusions

This study developed a data-driven multi-objective optimization framework to efficiently refine the composition of 3D-printable CSA cement composites. The key findings are:

(1) Surrogate model: A Bayesian-optimized BP neural network with a bootstrap ensemble provides accurate predictions (test R^2^ ≥ 0.913) and quantifies uncertainty via 95% confidence intervals.
(2) Algorithm efficiency: Over 30 independent runs, the NSWOA reaches a hypervolume of 7.515 after only 20 generations—virtually identical to the 7.500 achieved by NSGA-II after 100 generations. Thus, the NSWOA is highly sample-efficient and well-suited for limited iteration budgets.
(3) Experimental success: Starting from seven primary mixtures and 28 initial samples, the framework identified 23 Pareto-optimal formulations within 20 iterations. Five randomly selected formulations were experimentally validated with prediction errors below 5%.
(4) Materials insights: Optimal formulations minimize retarder-rich mixtures (I, II) and favor bentonite- and PP fiber-rich ones (V, VII). The synergy between bentonite (enhancing thixotropy) and PP fibers (improving strength) is highlighted.
(5) Limitations and outlook: The small initial dataset and laboratory-scale specimens constrain generalizability. Future work will expand the dataset, test more Pareto-optimal formulations, and incorporate durability and cost criteria.

The workflow demonstrates that machine learning-assisted optimization can accelerate formulation refinement under limited experimental budgets. Code and data are provided for reproducibility.

## Figures and Tables

**Table 1:** Base ingredients and mix proportions of 3D-printed mortars.

Formulation Ingredients			Primary Formulations						
Specimens			I	II	III	IV	V	VI	VII
Raw materials	Water		35%						
	WRA		0.3%						
	UQS		1.0%						
	Cementitious materials	CSA	100%						
	Thickeners	HPMC	0.4%						
	Rheology modifier	Diatomite	-	-	6.0%	-	-	-	-
		Bentonite	-	-	-	-	2.0%	-	1.0%
		Metakaolin	-	-	-	3.0%	-	-	-
		PMS	-	-	-	-	-	10.0%	-
	Retarder	TA	0.2%	-	-	0.15%	-	0.25%	0.2%
		SG	-	0.05%	0.2%	-	0.2%	-	-
	Fiber	PP	-	-	-	-	-	-	0.75%
Performance	Thixotropy	Structure parameters	0.115	0.174	0.154	0.188	0.246	0.376	0.1
	Mechanical strength	Compressive strength (MPa)	34.0	32.5	37.0	30.0	30.6	27.0	40.0
	Shape stability	Structure deformation (%)	9.0	9.9	6.9	4.9	3.9	7.5	6.0

**Table 2:** Chemical compositions of CSA cement (% by mass).

Components	Al_2_O_3_	SiO_2_	SO_3_	CaO	Fe_2_O_3_	MgO	TiO_2_	Na_2_O	Others
CSA cement	22.14	8.98	16.76	45.67	2.31	2.46	1.13	0.07	0.48

**Table 3:** Physical properties of PP fiber (provided by manufacturer).

Fiber	Density (g/cm^3^)	Tensile Strength (GPa)	Young’s Modulus (GPa)	Length (mm)	Filament Diameter (μm)	Rupture Elongation (%)
PP	0.91	0.5	4.5	6	50	20 ± 5

**Table 4:** Mix proportions and target values of 28 initial experimental samples.

	I	II	III	IV	V	VI	VII	Structure Parameters	Compressive Strength (MPa)	Structure Deformation (%)
1	1	0	0	0	0	0	0	0.115	34	9
2	0	1	0	0	0	0	0	0.174	32.5	9.9
3	0	0	1	0	0	0	0	0.154	37	6.9
4	0	0	0	1	0	0	0	0.188	30	4.9
5	0	0	0	0	1	0	0	0.246	30.6	3.9
6	0	0	0	0	0	1	0	0.376	27	7.5
7	0	0	0	0	0	0	1	0.1	40	6
8	0.5	0	0.5	0	0	0	0	0.14	35.5	7.5
9	0.5	0	0	0.5	0	0	0	0.165	31	6.5
10	0.5	0	0	0	0.5	0	0	0.18	32	5.5
……	…	……	…	…	…	…	…	……	……	……
21	0	0	0	0	0	0.5	0.5	0.28	36	6.8
22	0	0	0.3	0	0	0.3	0.4	0.2	35.2	6.7
23	0	0	0	0.4	0	0.4	0.2	0.25	31	6.16
24	0	0	0	0	0.3	0.4	0.3	0.254	32	5.97
25	0	0	0.5	0	0	0.5	0	0.26	32	7.2
26	0	0	0	0.5	0	0.5	0	0.28	28.5	6.25
27	0	0	0	0	0.5	0.5	0	0.31	28.7	5.8
28	0	0	0.3	0.3	0.4	0	0	0.2	32.3	5.1

**Table 5:** Comparison between predicted and experimental values for selected samples from Pareto front.

	I	II	III	IV	V	VI	VII	Structure Parameters			Compressive Strength (MPa)			Structure Deformation (%)		
								Pred	Real	Error	Pred	Real	Error	Pred	Real	Error
1	0	0	0.01	0.07	0.13	0	0.79	0.127	0.125	1.6%	38.81	38.05	2.0%	5.92	5.66	4.6%
2	0.01	0	0.05	0.04	0.22	0.02	0.66	0.14	0.143	2.1%	37.64	37.11	1.4%	5.77	5.61	2.9%
3	0	0.01	0.01	0	0.53	0	0.46	0.18	0.178	1.1%	35.27	34.97	0.9%	4.7	4.92	4.5%
4	0	0	0.15	0.01	0.56	0.08	0.19	0.21	0.21	0	33.15	33.11	0.12%	5.22	5.10	2.4%
5	0	0	0.03	0.01	0.50	0.30	0.16	0.28	0.285	1.8%	30.87	31.11	0.77%	5.33	5.42	1.7%

**Table 6:** Performance comparison of surrogate models (BO-BP, GPR, RF) on test set.

Model	Output	RMSE	R^2^
BO-BP	Structure parameters	0.0075	0.950
	Compressive strength (MPa)	0.4641	0.913
	Structure deformation (%)	0.0841	0.982
GPR	Structure parameters	0.0088	0.930
	Compressive strength (MPa)	0.3804	0.942
	Structure deformation (%)	0.2395	0.853
RF	Structure parameters	0.0459	−0.908
	Compressive strength (MPa)	2.4674	−1.459
	Structure deformation (%)	0.7121	−0.300

**Table 7:** Hypervolume (mean ± std) over 30 independent runs for NSGA-II and NSWOA at different generations.

Generation	NSGA-II	NSWOA	Wilcoxon *p*-Value
10	7.2485 ± 0.2186	7.6469 ± 0.2420	7.09 × 10^−8^
20	7.2713 ± 0.2117	7.5147 ± 0.1941	6.36 × 10^−5^
30	7.3228 ± 0.1857	7.4728 ± 0.1617	3.00 × 10^−4^
100	7.4998 ± 0.0624	7.3722 ± 0.0119	1.94 × 10^−10^

**Table 8:** Predicted vs. experimental values for four Pareto-optimal formulations selected from NSWOA (generation 20) and four from NSGA-II (generation 30).

	I	II	III	IV	V	VI	VII	Structure Parameters	Compressive Strength (MPa)	Structure Deformation (%)	Avg Err
NSWOA	0.04	0	0	0.07	0.21	0.02	0.66	0.14	37.50	5.66	0.8%
	0.05	0	0.05	0	0.54	0	0.36	0.18	34.48	4.95	0.73%
	0	0	0.07	0	0.15	0.27	0.51	0.20	35.40	6.24	0.97%
	0.03	0.01	0.03	0	0.50	0.25	0.18	0.24	31.73	5.62	0.84%
NSGA-II	0.03	0.12	0.29	0.05	0.07	0	0.44	0.14	37.28	6.59	26.49%
	0	0.03	0	0.32	0.11	0.09	0.45	0.17	34.47	5.78	14.73%
	0.02	0.02	0.25	0.14	0.12	0.20	0.25	0.20	34.13	6.28	9.7%
	0	0.03	0.02	0.31	0.24	0.29	0.11	0.24	30.46	5.88	12.07%

## Data Availability

The original contributions presented in this study are included in the article. Further inquiries can be directed to the corresponding authors.
